# Phototropin2 LOV1 plays a role in strong light signal transduction leading to chloroplast avoidance response

**DOI:** 10.1007/s11738-026-03904-x

**Published:** 2026-05-12

**Authors:** Weronika Krzeszowiec, Lilian Nehlin, Nikola Winter, Andreas Bachmair, Sebastian Pintscher, Halina Gabryś

**Affiliations:** 1 Department of Plant Biotechnology, Faculty of Biochemistry, Biophysics and Biotechnology, Jagiellonian University, Gronostajowa 7, Krakow, 30-387 Poland; 2 Department of Biochemistry and Cell Biology, Max Perutz Labs, University of Vienna, Dr. Bohr Gasse 9, Vienna, 1030 Austria

**Keywords:** Avoidance, Blue light, Chloroplast movements, LOV domain, Phototropin, Ubiquitin

## Abstract

In a recent study on the role of the ubiquitin-proteasome system in seedling and chloroplast development, a ubiquitin variant ubK48R was inducibly expressed in *Arabidopsis thaliana*, (Talloji et al. 2022). In addition to inhibiting proteolysis, its expression during germination caused both defects in chloroplast development and seedling death. This seedling lethality could be suppressed by mutating PHOT2, the gene that encodes the blue light receptor, phototropin2. The aim of this study was to establish how the mutations of phototropins introduced into the Arabidopsis genome to counteract the lethal effect of UbK48R expression affect phototropin-mediated chloroplast responses to blue light. We compared blue-light-controlled chloroplast redistribution in the leaves of several phototropin mutants, in particular in two frameshift mutants and a deletion mutant of PHOT2 with three amino acids deleted in the photosensory LOV1 domain. The deletion mutant showed decreased functionality of the photoreceptor protein, which was manifested in a marked decrease in the chloroplast avoidance response. A comparison of AlphaFold3 predicted structural models for the wild type and mutated PHOT2 proteins revealed that the lack of the three amino acids disrupts interdomain interactions near the deletion site and affects a potential dimerization interface. Our results point to an important function of the PHOT2 LOV1 domain in light signal transduction, which results in chloroplasts avoiding excess light.

**Supplementary Information:**

The online version contains supplementary material available at 10.1007/s11738-026-03904-x.

## Introduction

Plants use several photoreceptors to assess the quality, quantity and direction of light. Whereas phytochromes absorb light in the red and far-red regions of the spectrum, and UVR8 is specific to UV-B, all other photoreceptors absorb blue light. Among these, phototropins are unique in that they are plasma membrane-associated (Sakamoto and Briggs 2002; Christie et al. 2011; Ishishita et al. 2020). This attribute enables them to detect light direction (Zurzycki 1980) so that they can modulate growth and control organelle movement direction on the basis of light intensity. Higher plants have two types of phototropins (phot1 and phot2), distinguished by their light sensitivity, and, as a consequence, by the processes they regulate (Briggs and Christie 2002). Both phototropins share the same general structure, consisting of an N-terminal light-sensitive domain followed by a C-terminal serine/threonine kinase domain. Each light-sensitive domain consists of two blue light-receiving, FMN-binding LOV1 and LOV2 (light-oxygen-voltage –sensing) domains. The kinase domain is responsible for transmitting the light-generated signal inside the cell (Cho et al. 2007).

Phototropins cooperate by regulating phototropism and chloroplast movement responses, which enable plants to optimize the conditions of photosynthesis (Sakai et al. 2001; Aihara et al. 2008), for review see (Łabuz et al. 2022). Phot1 contributes to maximizing the absorption of low intensity light by controlling chloroplast accumulation in weak blue light (wBL). Phot2 is less sensitive, but indispensable for essential light-dependent processes, such as chloroplast re-localization in response to strong blue light (SBL). Interestingly, a fraction of this protein is deemed to reside on the chloroplast envelope and detect local light exposure, even though the majority of the protein resides on the plasma membrane (Ishishita et al. 2020).

Recent work using the inhibition of the ubiquitin-proteasome system (UPS) indicates that light signal transduction depends on protein turnover, and phototropin 2 might be critically affected by mis-regulation of the UPS. In particular, this study analyzed the role of the UPS in the *Arabidopsis thaliana* seedling and chloroplast development (Talloji et al. 2022). The ubiquitin variant ubK48R was inducibly expressed in an *Arabidopsis thaliana* plant line which carried the *UbK48R* transgene (RV85-6). The expression was found to inhibit proteolysis via the UPS and to negatively affect various developmental processes. Expression of ubK48R during germination caused defects in chloroplast development, which, as a consequence, led to seedling death. Mutations in three different single genes were found to suppress seedling lethality. One of these genes was *PHOT2*, which encodes the blue light receptor, phototropin2 (phot2). To inquire into the basis of lethality suppression, the authors investigated whether mutations in phototropins have impact on seedling survival after ubK48R induction. Mutations in PHOT2 were generated using the CRISPR/Cas9 system in the RV86–5 background. Two frameshift mutations, namely, a one base pair deletion (*phot2–81*), a two base pair deletion (*phot2–82*), and an in-frame, nine base pair deletion allele (*phot2–83*) were generated. Of these three mutations, only the latter, nine base pair (three amino acid) deletion mutation in *PHOT2*, demonstrated a significantly stronger suppressing power. Both de-etiolation and seedling survival were supported. On the contrary, a T-DNA insertion mutation in *PHOT1* appeared to promote death of RV86–5 seedlings with ubK48R induced during germination (Talloji et al. 2022).

The major function of phot2 in vascular plants is to mediate chloroplast avoidance of excess light by responding to strong blue light (SBL) (Jarillo et al. 2001). Therefore, we asked the question as to how the mutations introduced into the *A.thaliana* genome to counteract the lethal effect of *UbK48R* expression affect chloroplast responses to blue light mediated by phototropins.

In this study, we compare two frameshift alleles of PHOT2 with a three amino acid deletion allele in terms of light-dependent chloroplast movement. We show that the deletion allele decreases the functionality of the protein and affects a potential dimerization interface. The experiments point to an important function of the PHOT2 LOV1 domain in light signal transduction.

## Materials and methods

### Plant material

The plants used in this study were *Arabidopsis thaliana* accession Col-0 (AthWT), *phot1*, *phot2*,* phot1phot2*, and lines generated in the UPS study: RV86-5, *phot1-1*, *phot2-81*, *phot2-82*, *phot2-83* as well as two double mutants, *phot1-1phot2-82* and *phot1-1phot2-83*.

*Arabidopsis thaliana* (L.) Heynh. Columbia-0 and *phot1* (SALK_088841) seeds were purchased from the Nottingham Arabidopsis Stock Centre (NASC, Nottingham, UK). *phot2* seeds were a kind gift from José Jarillo. The *phot1phot2* double mutant was obtained by crossing *phot1* and *phot2* plants.

Plant line RV86-5 contains a Dexamethasone-inducible transgene for the high-level expression of a ubiquitin variant with K48R change, to decrease formation of Lys 38-linked ubiquitin chains. It has the Col-0 background and does not express the transgene in the absence of Dexamethasone. This line has been described previously (Schlögelhofer et al. 2006; Talloji et al. 2022).

Plants were grown in peat pellets (Jiffy International AS) in a growth chamber (Sanyo MLR-350 H) at 23 ± 2 °C, 80% relative humidity, with a 10 h light /14 h darkness photoperiod. They were illuminated with fluorescent lamps (Philips Master TL-D-36 W/840, Osram L36 W/77 Fluora, Activa 172 –36 W, Sylvania Gro-Lux F36W/GRO-T8), at an average fluence rate of 100 ± 20 µmol m^− 2^ s^− 1^. In all experiments, mature leaves of four- to six-week-old plants were used.

### Photometric assessment of chloroplast movements

Quantitative measurements of chloroplast movements were taken from detached leaves or leaf segments using a double-beam photometer (Gabryś et al. 2017). A red light of 660 nm, 0.1 µmol m^− 2^ s^− 1^, modulated with a frequency of 800 Hz was used to monitor changes in transmittance through the leaf. Chloroplast movements were elicited using blue light of 460 nm (Luxeon Royal Blue LXHL-FR5C diode, Philips Lumiled Lighting Comp., San Jose, CA, USA).

Prior to the assessment of chloroplast movement, the plants were dark-adapted overnight for at least 12 h. Leaves with initial transmittance levels differing by less than 1% were chosen for the experiments. The experiments were performed in two modes. (1) The leaf segments were illuminated perpendicular to the adaxial surface of the blade with wBL of 1.4 µmol m^− 2^ s^− 1^ for 45 min followed by SBL of 108 µmol m^− 2^ s^− 1^ for the same time. Two types of parameters were measured/calculated for both responses: amplitude – the transmittance change after 45 min, and velocity – the first derivative of the initial linear fragment of the transmittance curve (for details see (Gabryś et al. 2017). (2) To measure the fluence rate-response curves, chloroplast relocations were elicited using the continuous blue light of fluence rates increasing stepwise, each step lasting 1 h. In these experiments, all amplitudes were measured against the initial, dark transmittance level. For practical reasons, only two velocities were calculated: one, of the first accumulation response to 0,07 µmol m^− 2^ s^− 1^, and the second, of the first stable avoidance response at 18 µmol m^− 2^ s^− 1^.

#### Statistical analysis

Parameters of chloroplast responses are presented as means with standard error. A one-way analysis of variance (ANOVA) was performed, followed by a post hoc Tukey Kramer test (multiple comparisons of means) to determine differences between the means. The statistical analyses were performed with GraphPad InStat version 3.10.

### Western blot

The seeds were germinated on MS agar plates supplemented with 1% sucrose. After two days in darkness at 4 °C, the plants were put to light (16 h light, 8 h dark) and grown for seven days. The plants were snap-frozen in liquid nitrogen and stored at -70 °C until protein extraction. The frozen plant material was ground in a TissueLyser (Quiagen) before buffer was added (90 mM HEPES pH 7.4, 4% SDS, 20 mM DTT, 20 µg/ml pepstatin, 1x protease inhibitor cocktail (Sigma P9599)). The extract was incubated for six minutes at 95 °C, then centrifuged for 10 min. The supernatant was transferred to a new tube and supplemented with SDS loading dye. Proteins were separated on a 10% SDS gel and subsequently blotted to a PVDF membrane (60 min, 100 V, 4 °C). These proteins were visualized using BioRad stain-free imaging. After blocking the membrane with dried skimmed milk, primary antibodies (anti-PHOT1: Agrisera AS10 720 in 1:5000 dilution and anti-PHOIT2: Agrisera AS10 721 in 1:10000 dilution, and control antibody (anti-Actin: Agrisera AS16 3140 in 1:2000 dilution) were used to visualize specific proteins. The secondary antibody was anti-rabbit HRP (1:5000 dilution) for detection with Advansta Western Bright Sirius substrate, and imaging was performed using a BioRad ChemiDoc MP system.

### Protein modelling

Predictions of protein structure and interactions between domains for WT and mutant PHOT2 LOV were carried out in AlphaFold3 (Abramson et al. 2024). AlphaFill was used to predict the binding sites for the FMN cofactors (Hekkelman et al. 2023). The sequence of the polypeptide chain of WT phot2 from *Arabidopsis thaliana* was taken from the UniProt database (entry: P93025).

## Results and discussion

### Chloroplast responses in novel phototropin mutants

The photometric method used in this study enables one to compare chloroplast movements only in leaves of the same mesophyll tissue geometry, e.g. cell shapes, number and size of chloroplasts etc. (Gabryś et al. 2017). The leaf cross-sections were examined microscopically to make certain that the comparison was justified for the mutant lines that were to be analyzed. Analysis of around 20 cross-sections per line, typical examples of which are shown in Fig. S1, did not reveal any differences that would make the comparison of chloroplast responses problematic.

**Fig. 1:**
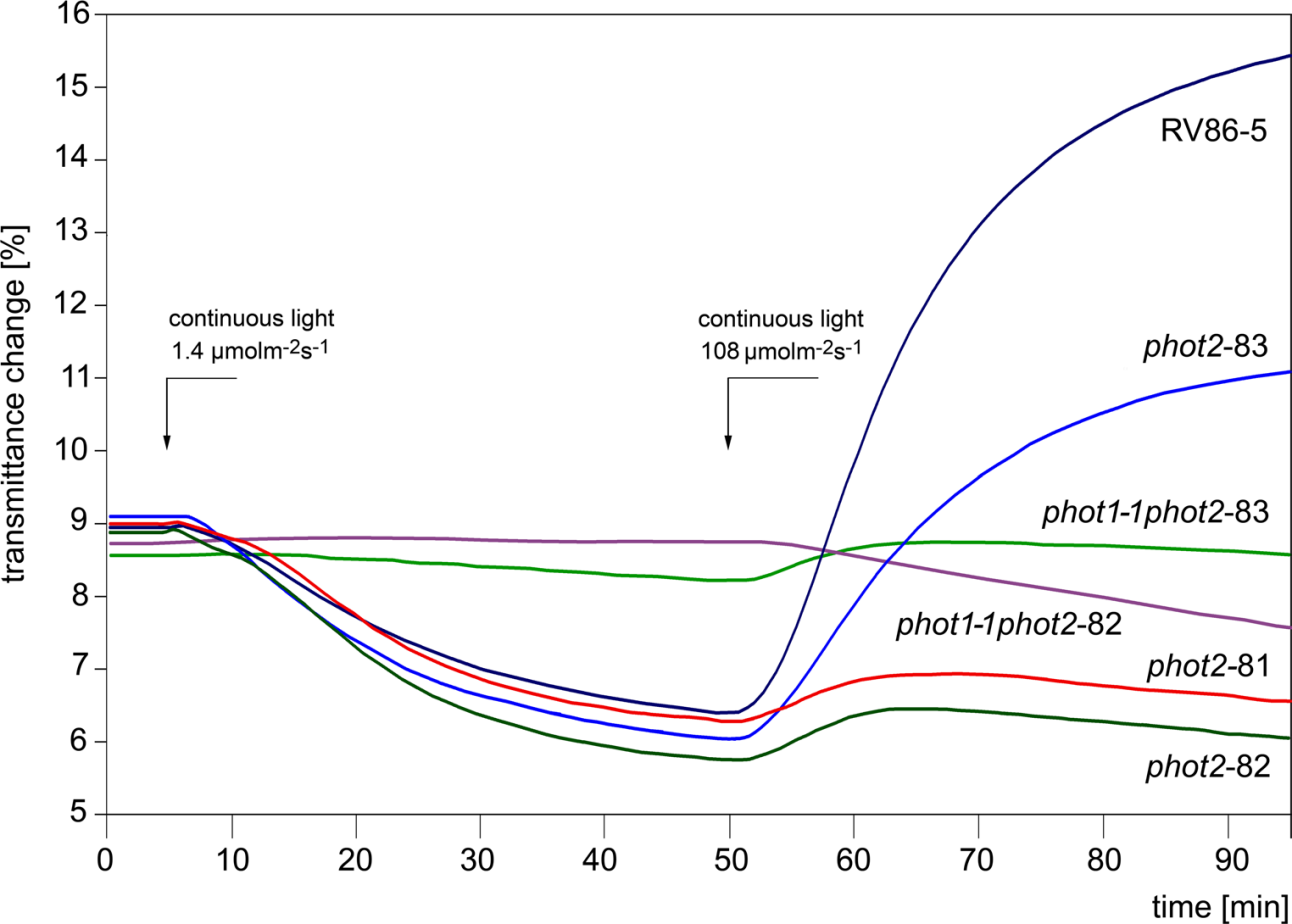
Blue light-activated chloroplast movements in single and double phototropin2 mutants. Average transmittance changes were measured in detached, dark-adapted leaves irradiated with continuous blue light saturating accumulation (transmittance decrease) and avoidance (transmittance increase) responses. Numbers of averaged traces are given in brackets: RV86-5 (43, navy blue), *phot2-81* (15, red), *phot2-82* (31, dark green), *phot2-83* (29, blue), *phot1-1phot2-82* (22, purple), *phot1-1phot2-83* (26, green)

The average transmittance changes shown in Fig. 1. reflect chloroplast redistribution in response to wBL followed by SBL in leaves of the three *phot2* mutants labelled *81*, *82* and *83* and two double mutants: *phot1-1 phot2-82* and *phot1-1 phot2-83*.

**Fig. 2:**
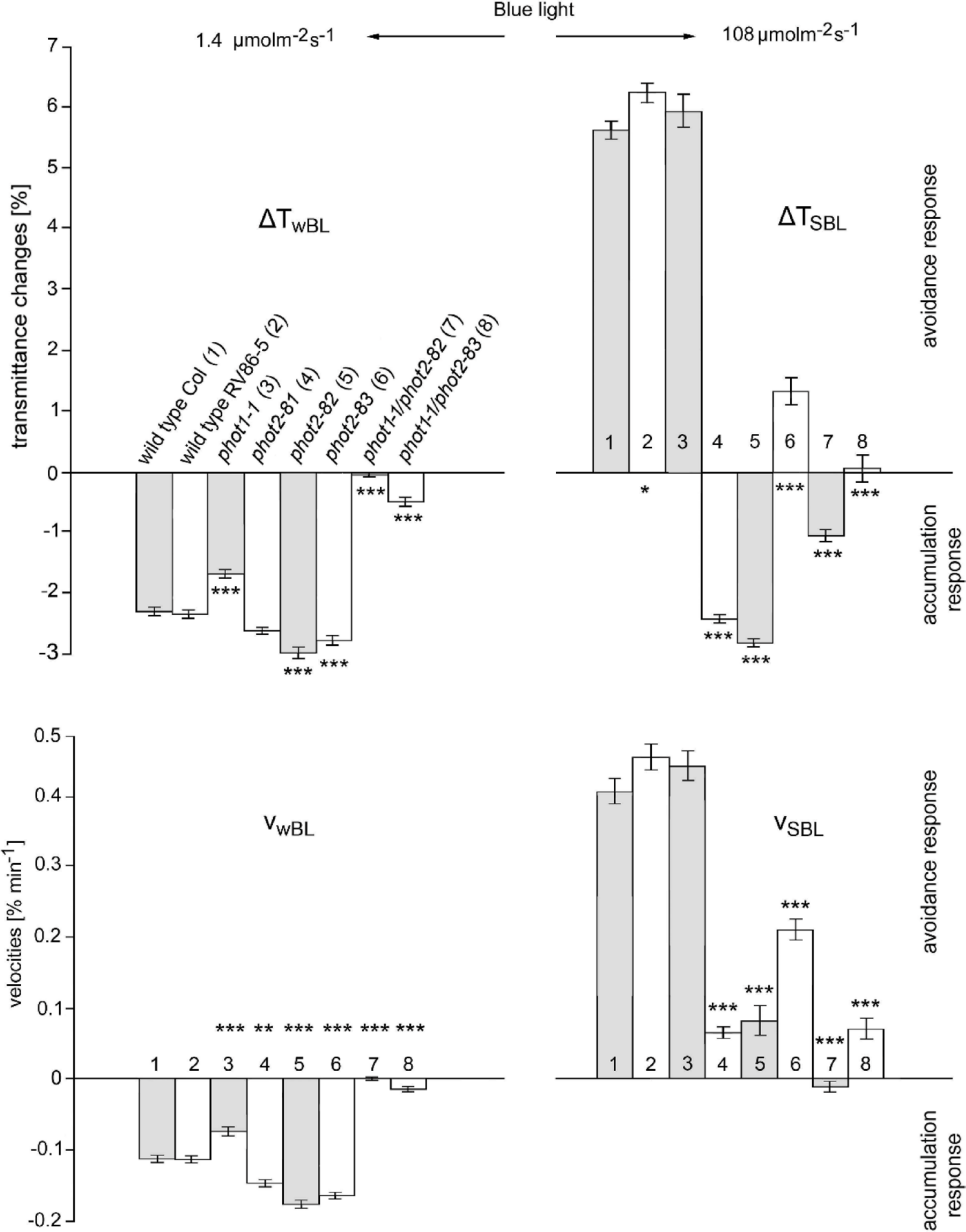
Parameters of transmittance changes accompanying chloroplast responses to continuous blue light. ∆T_wBL_ – decrease in leaf transmittance after 45 min irradiation with wBL; ∆T_SBL_ - increase in leaf transmittance after 45 min irradiation with SBL; v_wBL_ and v_SBL −_ the respective velocities of transmittance changes measured as the first derivatives of the initial, quasi-linear parts of the curves. Numbers of averaged parameters range from 43/41 for WT/ RV86-5, 27/26 for *phot2-83/phot2-82*, 20 for *phot1phot2* and *phot1phot2-83* to 16 for *phot1-1* and *phot2-81*. The results represent the means of three biological replicates with error bars denoting standard deviation (SD). Asterisks indicate the statistical significance of the difference between the parameters of chloroplast responses measured in genetically modified lines and in wild-type Columbia leaves. The statistical analysis was performed using the Tukey-Kramer Multiple Comparisons Test

**Fig. 3:**
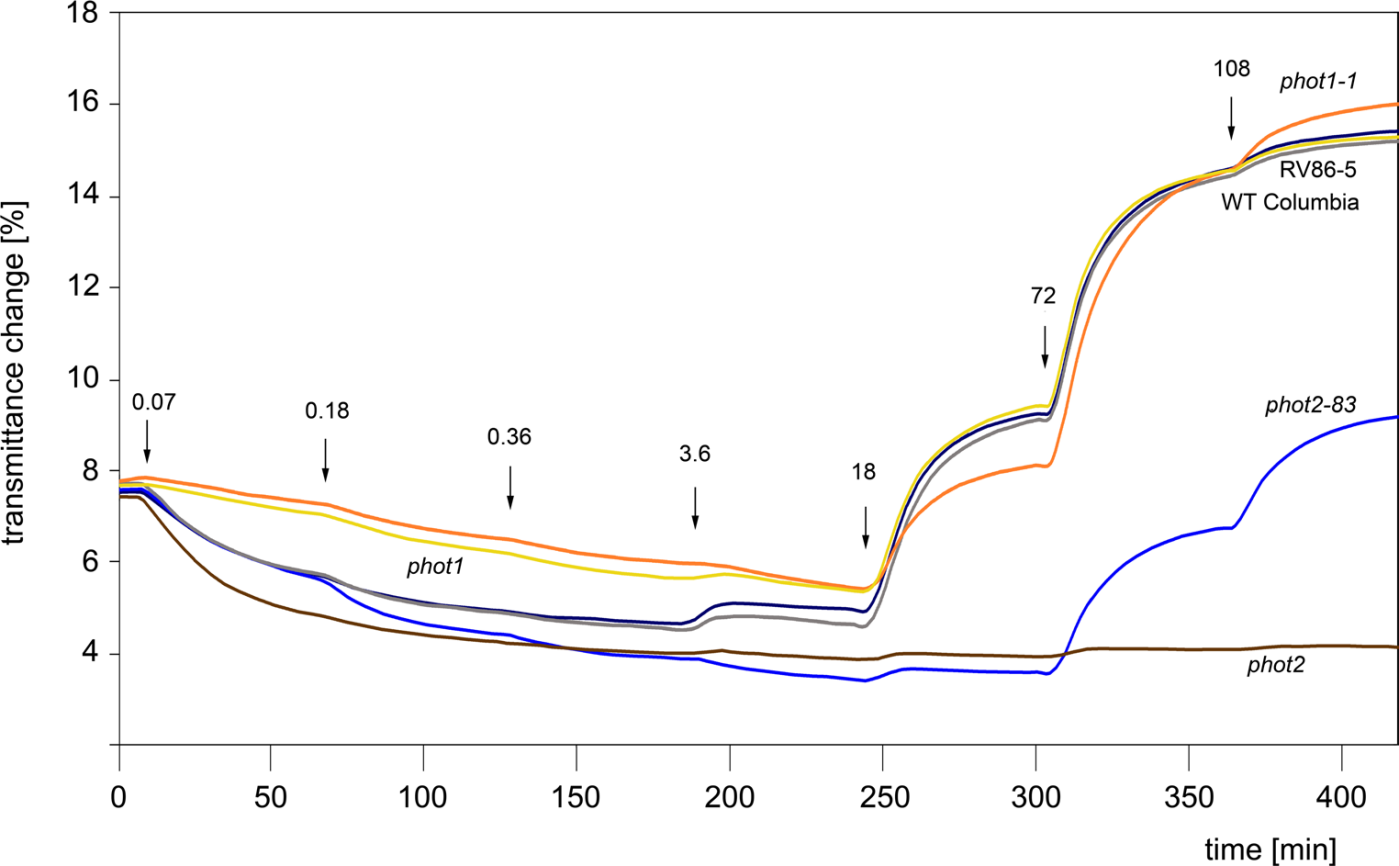
Fluence-rate-response curves for blue light-activated chloroplast movements in leaves of wild type plants (WT Columbia and RV86-5) and phototropin mutants (*phot1*, *phot1-1*, *phot2* and *phot2-83*). Leaves of dark-adapted plants were irradiated with continuous blue light of intensity increased in 1 h-long steps. Fluence-rates in µmol quanta m^−2^s^− 1^ are given at the arrows that mark each step onset. Numbers of averaged traces are given in brackets: WT(9, gray), RV86-5 (7, navy blue), *phot1* (9, yellow), *phot1-1* (10, orange), *phot2* (10, brown), *phot2-83* (16, blue)

The parameters which characterize these responses, transmittance amplitudes and velocities, are given in Fig. 2. As all three *phot2* mutant alleles were obtained in the genetic background of RV86-5, the latter line served as a control. Comparison of the parameters measured for WT Columbia and RV86-5 did not reveal differences in the accumulation responses; a slight, statistically relevant difference was noted only between the amplitudes of avoidance responses (Fig. 2, bars 1 and 2). Likewise, fluence rate-response curves for the WT Columbia and RV86-5 lines, shown in Fig. 3, are practically superimposed.

In weak light, chloroplasts of all three single mutants exhibited very similar accumulation responses (statistically relevant differences were found neither between amplitudes nor velocities). In contrast, they responded to strong light in a very different manner (Figs. 1 and 2, bars 4, 5 and 6). Both frameshift mutants, *phot2-81* and *phot2-82*, behaved similarly to the conventional *phot2* loss-of-function mutant (cf. also Fig. 3, bottom curve), which responded transiently to SBL. Instead, the chloroplasts of *phot2-83* exhibited a partial avoidance response with a total average ΔT of 5.1% as compared with 8.9% for the control plants. Notably, comparison of bars no. 2 and 6 in Fig. 2 for control and *phot2-83* leaves, reveals that amplitude and velocity are similarly affected by the three amino acid deletion in phot2.

The differences in chloroplast responses were also maintained in double mutants. The chloroplasts of the *phot1-1phot2-83* plant displayed residual accumulation and avoidance responses in wBL and SBL respectively. In contrast, the chloroplasts of *phot1-1phot2-82* did not react to wBL and, instead of avoidance, displayed distinct accumulation in SBL (Fig. 1 – middle curves, Fig. 2 – bars 7 and 8).

The results of the standard test commented on so far were obtained with wBL and SBL fluence rates saturating the wild type accumulation and avoidance responses respectively. However, under saturating light conditions, any possible differences between the lines measured may be flattened or even lost. Therefore, fluence rate-response dependencies were measured for the leaves of selected lines. The results are summarized in Figs. 3 and S2. A noteworthy feature of the *phot2-83* fluence rate-response curve is the transition from the accumulation amplitude which is identical to the WT/ RV86-5 control plants at the lowest light intensity, through the significantly higher accumulation amplitude, typical of the conventional *phot2* mutant at intermediate intensity steps, to the clear avoidance response at strong light steps. A distinct avoidance response of *phot2-83* starts at 72 µmol m^− 2^ s^− 1^, the fluence rate, at which amplitudes for WT/ RV86-5 chloroplasts reach over 92% of saturation.

As the double mutants had been obtained with *phot1-1*, we compared average fluence rate-response curves for both phototropin1 mutants (Fig. 3 and Fig. S2, bars c and d). A statistically significant difference between the two curves was noted only for the response to 18 µmol m^− 2^ s^− 1^ BL. At that fluence rate, chloroplasts dramatically change the direction of movement in the cell and begin the avoidance response. This reorientation, integrated over several cell layers, leads to the largest scatter in the tissue transmittance curves. As expected for the plants expressing no functional phot1, the amplitudes of the first three accumulation steps were around 1.5% lower in both mutants as compared to WT and/or RV86-5 plants, and the difference diminished at the fourth step to below 1% transmittance. At higher fluence rates (36 and 108 µmol m^− 2^ s^− 1^), the responses of both *phot1* mutants reached the level of those recorded for WT/ RV86-5, in accordance with phot2 being responsible for the avoidance responses of chloroplasts.

### Detection of mutated phototropins in leaf tissue

The levels of the proteins PHOT1 and PHOT2 were examined using Western blot in all investigated lines based on the RV86–5 background. As shown in Fig. S3 PHOT1 is not detectable in *phot1-1* single and double mutants, nor is PHOT2 in *phot2-81*,* phot2-82* and *phot2-83* single and double mutants. We nonetheless believe that a low level of the phot2-83 protein is present in the cells, but below the detection level of this blot, because a faint PHOT2 band was detected previously in experiments with seedlings (cf. ref. in (Talloji et al. 2022).

**Fig. 4:**
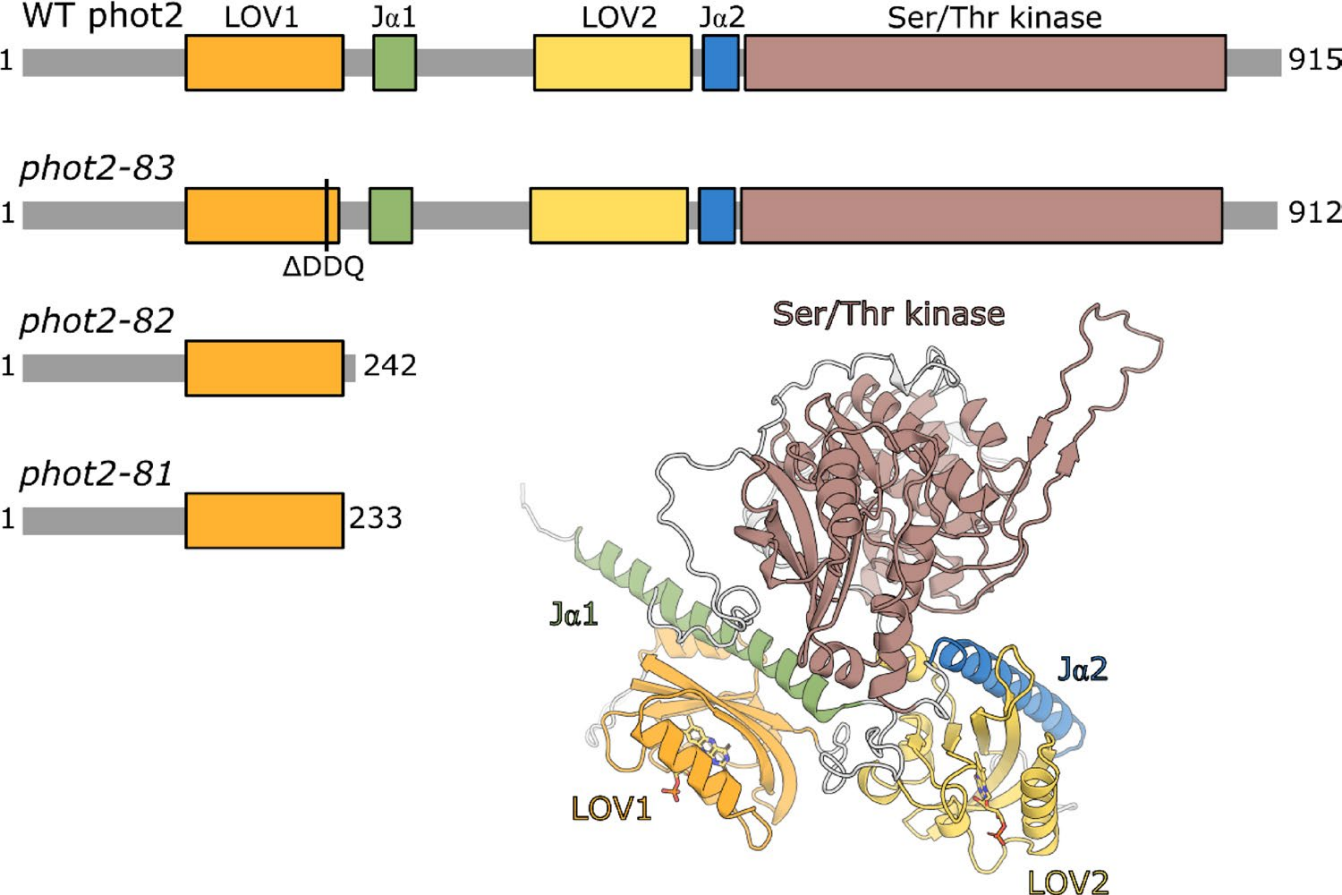
Structure of WT phototropin2 and its variants from this study. Block schemes represent domain organization in WT and variants. The 3D model represents an AlphaFold3 + AlphaFill predicted WT phot2 structure

**Fig. 5:**
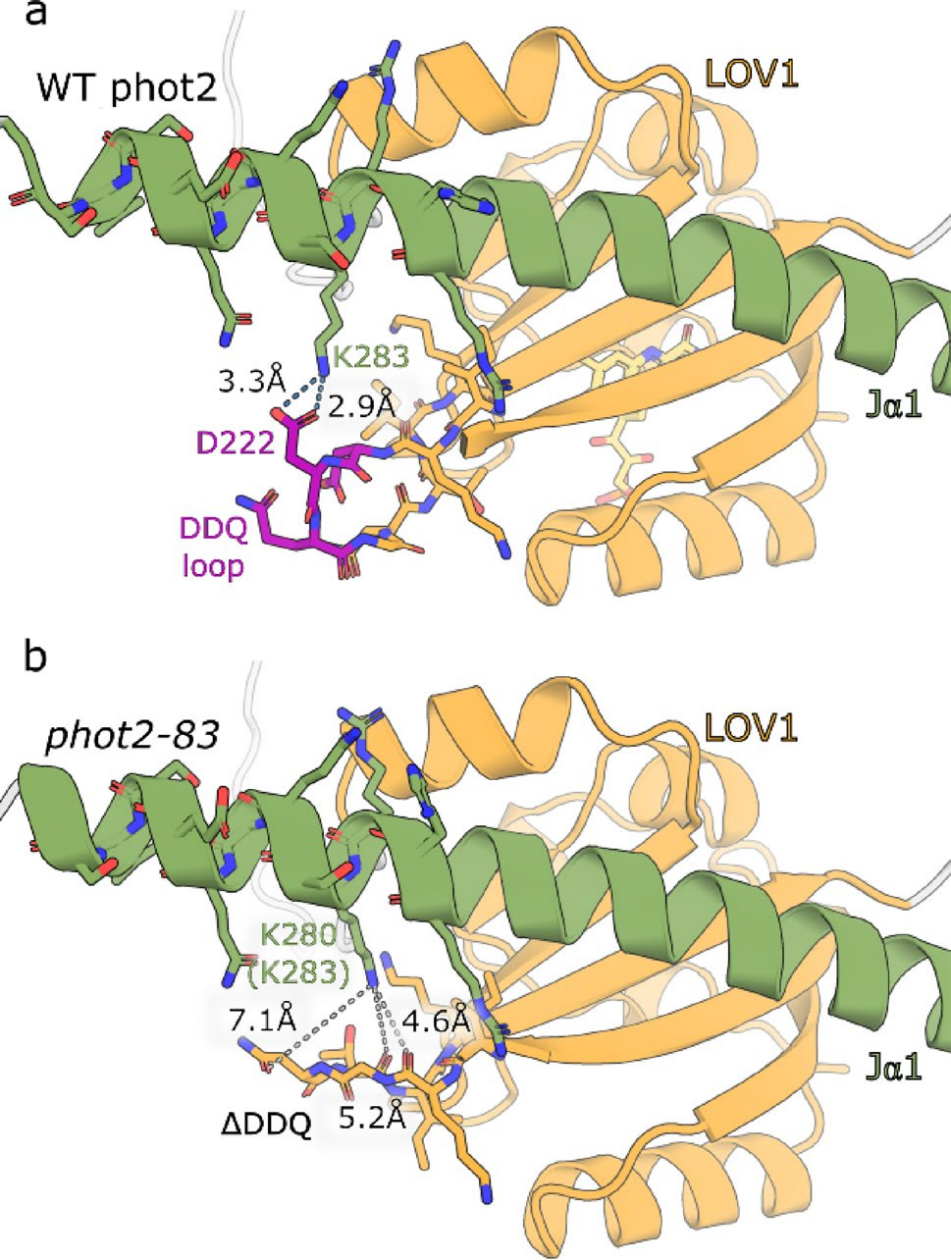
Interactions between Jα1 helix and LOV1 domain as predicted by AlphaFold3. In the case of WT (panel **A**), K283 can form a salt bridge to D222 from the DDQ loop (in purple). This salt bridge cannot be formed in the *phot2-83* three amino acid deletion mutant (panel **B**, DDQ loop deletion). The closest possible H-bonding partner for K280 (K283 in WT) is predicted to be too far to allow a direct H-bond contact

**Fig. 6:**
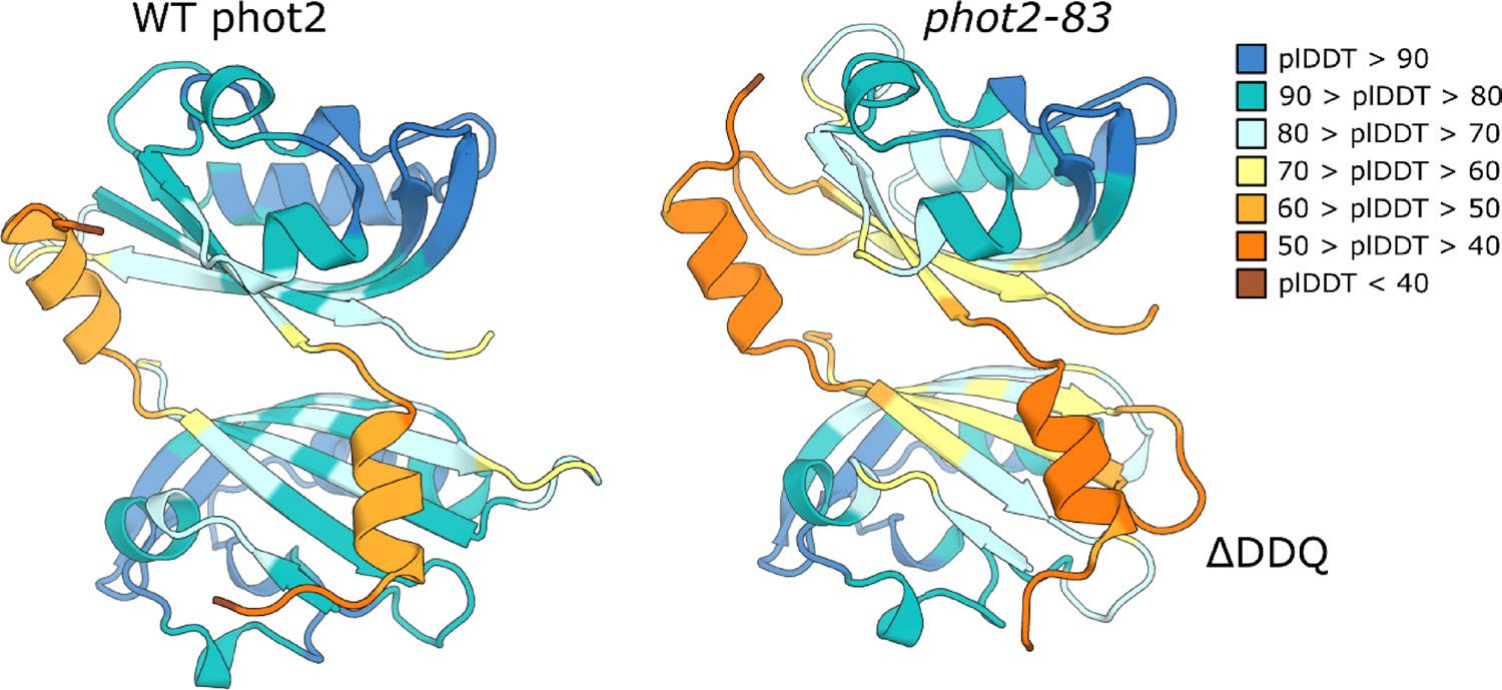
Dimerization of LOV1 domain in the WT phot2 and *phot2-83* variant as predicted by AlphaFold3. In the case of *phot2-83*, the model has lower plDDT scores at the dimerization interface, which indicates a less confident prediction

The mutation in *phot2-83*, the three amino acid deletion line generated with the CRISPR/Cas9 system, is known to affect the LOV1 (PAS1-PAC1) domain of phot2 (Talloji et al. 2022). This domain is essential for the photoreceptor’s functioning (Nakasako et al. 2008; Oide et al. 2018; Hart and Gardner 2021). Therefore, we decided to analyze the possible effects of this mutation in more detail by comparing the predicted structural models for the PHOT2 LOV1 domain in *phot2-83* versus WT using the tools AlphaFold3/AlphaFill. The results are presented in Figs. 4, 5 and 6 and Fig.S4.

### Protein structure prediction

The 3D model in Fig. 4 represents the predicted WT phototropin2 structure. Interestingly, AlphaFold3 predicted the existence of the Jα helix in the LOV1 domain. This possibility was not considered in the previous studies (see (Oide et al. 2018) and the references therein). We labelled this helix as Jα1. Structural models for the best-ranked predictions for WT phot2 and all *phot2* variants are shown in Fig. S4. The site of mutation in *phot2-83* is localized in the vicinity of the Jα1 helix, as shown in Fig. 5. This mutation leads to the deletion of three amino acids (DDQ, shown in purple), which are predicted to be a part of a larger loop in the WT (Fig. 5a). In the *phot2-83* mutant (Fig. 5b), their removal shortens the loop, altering its interactions with the neighboring residues, including the putative Jα1 helix. In the case of WT, K283 from Jα1 may form a salt bridge to D222 from the DDQ loop. This salt bridge cannot be formed in the *phot2-83* mutant (DDQ deletion). The closest possible H-bonding partner for K280 (K283 in WT) is predicted to be too far to allow a direct H-bond contact.

Isolated LOV1 domains of both phototropins have been shown to form dimers (Salomon et al. 2004; Nakasako et al. 2008). These domains may also play a role in the dimerization of phototropins; in particular, phot2 dimerization has been reported to occur through its LOV1 domains. Studies on purified full-length phot2 and its mutants have suggested a contribution of LOV1 to the kinase activity and BL-dependent conformational changes in phot2 (Oide et al. 2018). Thus, disturbance in the LOV domain structure may have far-reaching consequences for the biological activity and stability of the photoreceptor. The *phot2-83* mutation is located in the region that forms a homodimerization interface, and the deletion of three amino acids may affect the interaction surface of the two monomers, potentially decreasing dimer stability. Therefore, we tested the dimerization of the LOV1 domain in the WT phot2 and *phot2-83* variant and predicted the dimer structure with AlphaFold3. The results are shown in Fig. 6. In the case of *phot2-83*, the model has lower plDDT scores at the dimerization interface, which indicates a less confident prediction. The plDDT cannot be treated as a confident predictor of the dimer stability, therefore, a lower score should be considered circumstantial – but not conclusive – evidence.

Frequently, non-assembled subunits of complexes have higher turnover rates than the same subunit as part of a larger complex. For phot2, decreased homodimer stability might increase the monomeric fraction, with direct effects on the abundance of the biologically active dimer, and indirect effects due to turnover, via decreased availability of the protein for dimer formation. These hypotheses need to be tested in future work by in vitro analysis of the mutant protein.

### Implications on the role of LOV1 PHOT2 domain in light signal transduction

Of the two types of phototropin LOV domains, LOV2 located closer to the C-terminal kinase has been studied more thoroughly (Okajima et al. 2012; Oide et al. 2016; Aumonier et al. 2022). As a consequence, both the role of LOV2 in activating the kinase, and the mechanism of this activation, have been elucidated to a considerable extent. The function of LOV1 in these processes, however, is much less understood.

A relatively minor change in the region of the LOV1 domain, distant from the flavin chromophore, produces dramatic changes in two diverse physiological processes, namely, the rescue of seedling lethality caused by abnormal UPS, and chloroplast response to light stress. The effect of the *phot2-83* mutation on the chloroplast avoidance response shown in this study points to the important role that LOV1 plays in light activation/signal transduction by phototropin2. It is clear that the plant *phot2-*83 provides a valuable research object, not only for examining the roles of the LOV1 domain in activating phototropin2 in other blue light-controlled movement responses (i.e. phototropism, stomatal opening), but also for elucidating the mechanisms of different category rescue processes in higher plants.

## Supplementary Material

Below is the link to the electronic supplementary material.

Supplementary Material 1

Supplementary Material 2

Supplementary Material 3

Supplementary Material 4

## Data Availability

Data will be available on request.

## References

[CR1] Abramson J, Adler J, Dunger J, Evans R, Green T, Pritzel A et al (2024) Accurate structure prediction of biomolecular interactions with AlphaFold 3. Nature 630:493–500. 10.1038/s41586-024-07487-w 38718835 10.1038/s41586-024-07487-w PMC11168924

[CR2] Aihara Y, Tabata R, Suzuki T, Shimazaki KI, Nagatani A (2008) Molecular basis of the functional specificities of phototropin 1 and 2. Plant J 56:364–375. 10.1111/j.1365-313X.2008.03605.x 18643969 10.1111/j.1365-313X.2008.03605.x

[CR3] Aumonier S, Engilberge S, Caramello N, von Stetten D, Gotthard G, Leonard GA, Royant A (2022) Slow protein dynamics probed by time-resolved oscillation crystallography at room temperature. IUCrJ 9:756–767. 10.1107/S2052252522009150 36381146 10.1107/S2052252522009150 PMC9634615

[CR4] Briggs WR, Christie JM (2002) Phototropins 1 and 2: versatile plant blue-light receptors. Trends Plant Sci 7:204–210. 10.1016/s1360-1385(02)02245-8 11992825 10.1016/s1360-1385(02)02245-8

[CR5] Cho HY, Tseng TS, Kaiserli E, Sullivan S, Christie JM, Briggs WR (2007) Physiological roles of the light, oxygen, or voltage domains of phototropin 1 and phototropin 2 in Arabidopsis. Plant Physiol 143:517–529. 10.1104/pp.106.089839 17085510 10.1104/pp.106.089839 PMC1761953

[CR6] Christie JM, Kaiserli E, Sullivan S (2011) Light Sensing at the Plasma Membrane. In: Murphy A, Schulz B, Peer W (eds) The Plant Plasma Membrane. Plant Cell Monographs, vol 19. Springer, Berlin, Heidelberg. 10.1007/978-3-642-13431-9_19

[CR7] Gabryś H, Banaś AK, Hermanowicz P, Krzeszowiec W, Leśniewski S, Łabuz J, Sztatelman O (2017) Photometric assays for chloroplast movement responses to blue light. Bio-protocol 7:e2310. 10.21769/BioProtoc.2310 34541077 10.21769/BioProtoc.2310 PMC8410286

[CR8] Hart JE, Gardner KH (2021) Lighting the way: Recent insights into the structure and regulation of phototropin blue light receptors. J Biol Chem 296:100594. 10.1016/j.jbc.2021.100594 33781746 10.1016/j.jbc.2021.100594 PMC8086140

[CR9] Hekkelman ML, de Vries I, Joosten RP, Perrakis A (2023) AlphaFill: enriching AlphaFold models with ligands and cofactors. Nat Methods 20:205–213. 10.1038/s41592-022-01685-y 36424442 10.1038/s41592-022-01685-y PMC9911346

[CR10] Ishishita K, Higa T, Tanaka H, Inoue S, Chung A, Ushijima T, Matsushita T, Kinoshita T, Nakai M, Wada M, Suetsugu N, Gotoh E (2020) Phototropin2 contributes to the chloroplast avoidance response at the chloroplast-plasma membrane interface. Plant Physiol 183:304–316. 10.1104/pp.20.00059 32193212 10.1104/pp.20.00059 PMC7210631

[CR11] Jarillo JA, Gabrys H, Capel J, Alonso JM, Ecker JR, Cashmore AR (2001) Phototropin related NPL1 controls chloroplast relocation induced by blue light. Nature 410:952–954. 10.1038/35073622 11309623 10.1038/35073622

[CR12] Łabuz J, Sztatelman O, Hermanowicz P (2022) Molecular insights into the phototropin control of chloroplast movements. J Exp Bot 73:6034–6051. 10.1093/jxb/erac271 35781490 10.1093/jxb/erac271

[CR13] Nakasako M, Zikihara K, Matsuoka D, Katsura H, Tokutomi S (2008) Structural basis of the LOV1 dimerization of Arabidopsis phototropins 1 and 2. J Mol Biol 381:718–733. 10.1016/j.jmb.2008.06.033 18585389 10.1016/j.jmb.2008.06.033

[CR14] Oide M, Okajima K, Kashojiya S, Takayama Y, Oroguchi T, Hikima T, Yamamoto M, Nakasako M (2016) Blue light-excited light-oxygen-voltage-sensing domain 2 (LOV2) triggers a rearrangement of the kinase domain to induce phosphorylation activity in Arabidopsis phototropin1. J Biol Chem 291:19975–19984. 10.1074/jbc.M116.735787 27484797 10.1074/jbc.M116.735787 PMC5025684

[CR15] Oide M, Okajima K, Nakagami H, Kato T, Sekiguchi Y, Oroguchi T, Sekiguchi T, Hikima T, Yamamoto M, Nakasako M (2018) Blue light-excited LOV1 and LOV2 domains cooperatively regulate the kinase activity of full-length phototropin 2 from Arabidopsis. J Biol Chem 293:963–972. 10.1074/jbc.RA117.000324 10.1074/jbc.RA117.000324 PMC5777267 29196607

[CR16] Okajima K, Kashojiya S, Tokutomi S (2012) Photosensitivity of kinase activation by blue light involves the lifetime of a cysteinyl-flavin adduct intermediate, S390, in the photoreaction cycle of the LOV2 domain in phototropin, a plant blue light receptor. J Biol Chem 287:40972–40981. 10.1074/jbc.M112.406512 23066024 10.1074/jbc.M112.406512 PMC3510800

[CR17] Sakai T, Kagawa T, Kasahara M, Swartz TE, Christie JM, Briggs WR, Wada M, Okada K (2001) Arabidopsis nph1 and npl1: blue light receptors that mediate both phototropism and chloroplast relocation. Proc Nat Acad Sci 98:6969–6974. 10.1073/pnas.101137598 11371609 10.1073/pnas.101137598 PMC34462

[CR18] Sakamoto K, BriggsWR (2002) Cellular and subcellular localization of phototropin 1. Plant Cell 14:1735. 10.1105/tpc.003293 10.1105/tpc.003293 PMC151461 12172018

[CR19] Salomon M, Lempert U, Rüdiger W (2004) Dimerization of the plant photoreceptor phototropin is probably mediated by the LOV1 domain. FEBS Lett 572:8–10. 10.1016/j.febslet.2004.06.081 15304315 10.1016/j.febslet.2004.06.081

[CR20] Schlögelhofer P, Garzon M, Kerzendorfer C, Nizhynska V, Bachmair A (2006) Expression of the ubiquitin variant ubR48 decreases proteolytic activity in Arabidopsis and induces cell death. Planta 223:684–697. 10.1007/s00425-005-0121-z 16200408 10.1007/s00425-005-0121-z

[CR21] Talloji P, Nehlin L, Hüttel B, Winter N, Černý M, Dufková H, Hamali B, Hanczaryk K, Novák J, Hermanns M, Drexler N, Eifler K, Schlaich N, Brzobohatý B, Bachmair A (2022) Transcriptome, metabolome and suppressor analysis reveal an essential role for the ubiquitin-proteasome system in seedling chloroplast development. BMC Plant Biol 22:183. 10.1186/s12870-022-03536-6 35395773 10.1186/s12870-022-03536-6 PMC8991883

[CR22] Zurzycki J (1980) Blue light-induced intracellular movements. In: Senger H (ed) The blue light syndrome. Springer Berlin Heidelberg, Berlin, Heidelberg, pp 50–68

