# Supplementary material for: Phototropin2 LOV1 plays a role in strong light signal transduction leading to chloroplast avoidance response
Source: Acta Physiol Plant. 2026 May 12;48(5):36. doi: 10.1007/s11738-026-03904-x (PMC13167847; doi:10.1007/s11738-026-03904-x)
Supplement: Supplementary file 1 — Supplementary Material 1 [file 11738_2026_3904_MOESM1_ESM.docx]

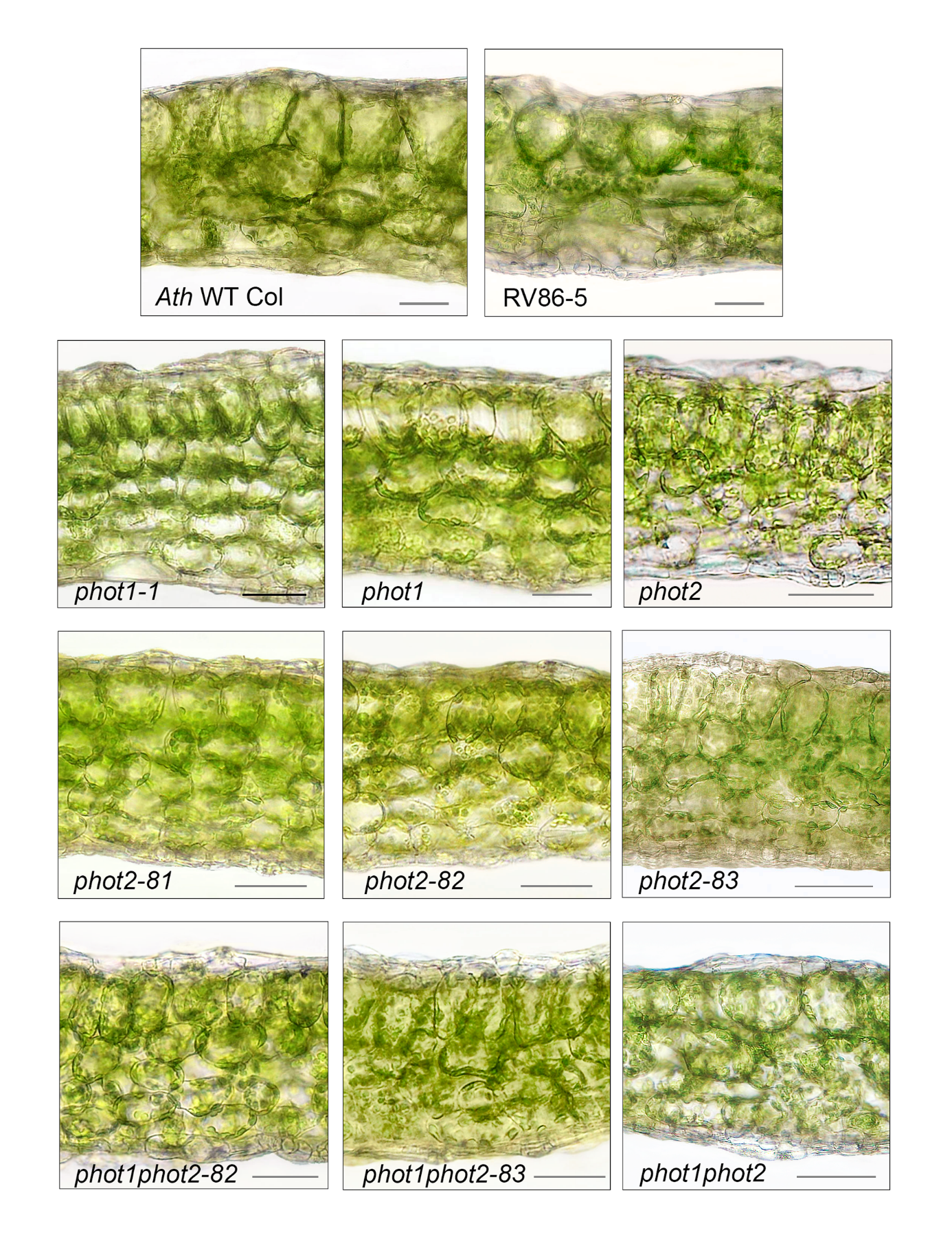


Fig.S1. Cross-sections of leaves of *A.thaliana* wild type and mutant lines used in this study. Bars on all figures = 50 µm.

Phototropin2 LOV1 plays a role in strong light signal transduction leading to chloroplast avoidance response;

W. Krzeszowiec, L. Nehlin, N. Winter, A. Bachmair, S. Pintscher, H. Gabryś; Acta Physiologiae Plantarum;

Corresponding author: Halina Gabryś, [halina.gabrys@uj.edu.pl](mailto:halina.gabrys@uj.edu.pl) ; Dept. of Plant Biotechnology, Faculty of Biochemistry, Biophysics and Biotechnology, Jagiellonian University, Gronostajowa 7, 30-387 Krakow, Poland
