# Supplementary material for: Phototropin2 LOV1 plays a role in strong light signal transduction leading to chloroplast avoidance response
Source: Acta Physiol Plant. 2026 May 12;48(5):36. doi: 10.1007/s11738-026-03904-x (PMC13167847; doi:10.1007/s11738-026-03904-x)
Supplement: Supplementary file 2 — Supplementary Material 2 [file 11738_2026_3904_MOESM2_ESM.docx]

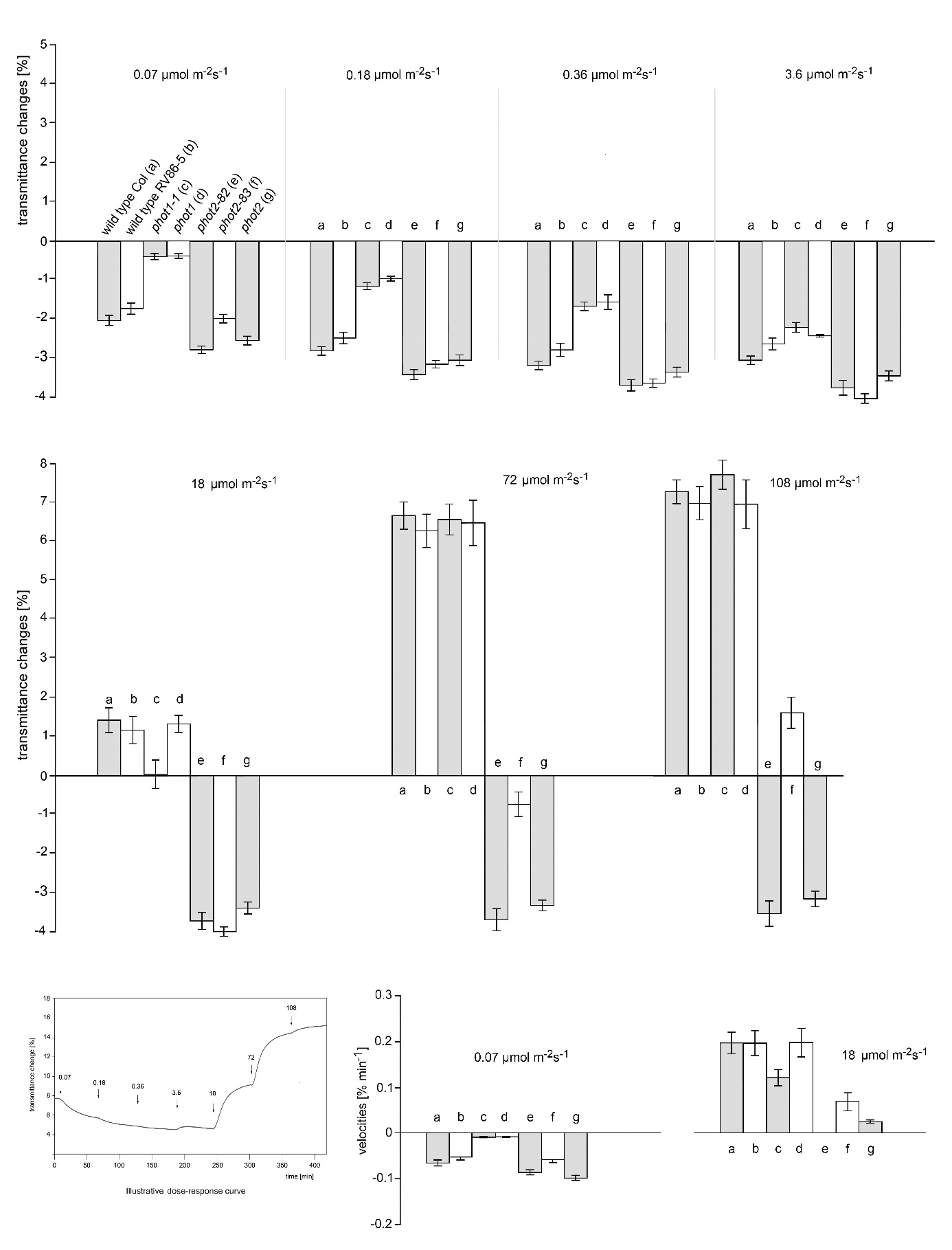


Fig.S2. The parameters of transmittance changes accompanying chloroplast responses to continuous blue light of intensity increased in 1h-long steps. An illustrative fluence-rate-response curve is shown bottom left. Fluence-rates in µmol quanta m^-2^s^-1^ are given above the sets of transmittance changes. Numbers of averaged parameters are given in brackets: WT(13), RV86-5 (12), *phot1-1* (14), *phot1* (10), *phot2-82* (4), *phot2-83* (16). Error bars denote standard deviation (SD).

Phototropin2 LOV1 plays a role in strong light signal transduction leading to chloroplast avoidance response;

W. Krzeszowiec, L. Nehlin, N. Winter, A. Bachmair, S. Pintscher, H. Gabryś; Acta Physiologiae Plantarum;

Corresponding author: Halina Gabryś, [halina.gabrys@uj.edu.pl](mailto:halina.gabrys@uj.edu.pl) ; Dept. of Plant Biotechnology, Faculty of Biochemistry, Biophysics and Biotechnology, Jagiellonian University, Gronostajowa 7, 30-387 Krakow, Poland
